# Finding Aquaporins in Annelids: An Evolutionary Analysis and a Case Study

**DOI:** 10.3390/cells10123562

**Published:** 2021-12-17

**Authors:** Serena Mucciolo, Andrea Desiderato, Marika Salonna, Tomasz Mamos, Viviane Prodocimo, Maikon Di Domenico, Francesco Mastrototaro, Paulo Lana, Carmela Gissi, Giuseppe Calamita

**Affiliations:** 1 Department of Invertebrate Zoology and Hydrobiology, University of Lodz, Banacha 12/16, 90-237 Lodz, Poland; andrea.desiderato@biol.uni.lodz.pl (A.D.); tomasz.mamos@biol.uni.lodz.pl (T.M.); 2 Centro de Estudos do Mar, Universidade Federal do Paraná, Av. Beira-Mar, s/n, Pontal do Sul, Pontal do Paraná 83255-976, PR, Brazil; didomenico@ufpr.br (M.D.D.); lana@ufpr.br (P.L.); 3 Institute of Medical Sciences, Foresterhill Health Campus, University of Aberdeen, Aberdeen AB25 2ZD, UK; marika.salonna@abdn.ac.uk; 4 Dipartimento di Bioscienze, Biotecnologie e Biofarmaceutica, Università degli Studi di Bari “A. Moro”, Via E. Orabona, 4, 70125 Bari, Italy; carmela.gissi@uniba.it (C.G.); giuseppe.calamita@uniba.it (G.C.); 5 Laboratório de Fisiologia Comparativa da Osmorregulação, Departamento de Fisiologia, Setor de Ciências Biológicas, Campus Politécnico, Universidade Federal do Paraná, Av. Cel. Francisco H. dos Santos 100, Curitiba 81531-980, PR, Brazil; vprodocimo@ufpr.br; 6 CoNISMa LRU, 70124 Bari, Italy; francesco.mastrototaro@uniba.it; 7 Dipartimento di Biologia, Università degli Studi di Bari “A. Moro”, 70124 Bari, Italy; 8 IBIOM, Institute of Biomembranes, Bioenergetics and Molecular Biotechnologies (IBIOM), CNR, Via Amendola 165/A, 70126 Bari, Italy

**Keywords:** annelids, aquaporin, evolution, *Alitta succinea*, estuarine invertebrate

## Abstract

Aquaporins (AQPs) are a family of membrane channels facilitating diffusion of water and small solutes into and out of cells. Despite their biological relevance in osmoregulation and ubiquitous distribution throughout metazoans, the presence of AQPs in annelids has been poorly investigated. Here, we searched and annotated *Aqp* sequences in public genomes and transcriptomes of annelids, inferred their evolutionary relationships through phylogenetic analyses and discussed their putative physiological relevance. We identified a total of 401 *Aqp* sequences in 27 annelid species, including 367 sequences previously unrecognized as *Aqps*. Similar to vertebrates, phylogenetic tree reconstructions clustered these annelid *Aqps* in four clades: AQP1-like, AQP3-like, AQP8-like and AQP11-like. We found no clear indication of the existence of paralogs exclusive to annelids; however, several gene duplications seem to have occurred in the ancestors of some Sedentaria annelid families, mainly in the AQP1-like clade. Three of the six *Aqps* annotated in *Alitta succinea*, an estuarine annelid showing high salinity tolerance, were validated by RT-PCR sequencing, and their similarity to human AQPs was investigated at the level of “key” conserved residues and predicted three-dimensional structure. Our results suggest a diversification of the structures and functions of AQPs in Annelida comparable to that observed in other taxa.

## 1. Introduction

Estuaries, including a large variety of intertidal environments, are highly dynamic systems characterized by large salinity fluctuations due to regular or stochastic events, such as pluviosity, river flows, tides, waves and storms [1]. Regular or irregular salinity variation is thus a major ecological and evolutionary challenge faced by organisms inhabiting these environments, which exhibit adaptations at different levels of biological organization. For instance, complex excretory systems and less permeable cuticles are common [2,3,4]. At the cellular level, membrane carrier and channel proteins work together to regulate osmolarity and cell volume. Carrier proteins, for instance, function to transport ions and neutral solutes through the plasma membranes. Among them, the Na-K-2Cl (NKCC) cotransporter and Na^+^/K^+^-ATPase are known to exert a central role in osmoregulation and cell volume homeostasis [5,6]. Water is the major component of biological fluids, and water movement into and out of cells is a property of life [7]. Driven by osmotic gradients, water can cross the plasma membrane through the phospholipid bilayer (simple diffusion) or by means of specific proteinaceous pores formed by aquaporin water channels, hereafter AQPs (facilitated diffusion) [8]. Water movement through AQPs is fast and can be regulated as it occurs during fluid secretion and absorption, and in cell volume homeostasis [7]. Membranes containing AQPs are five to 100 times more permeable to water than membranes lacking AQPs [9].

AQPs are a group of membrane channels widely expressed in nature, facilitating the transport of water and a number of small solutes, such as glycerol and other polyols, urea, hydrogen peroxide, ammonia and metalloids, as well as gases of biological relevance, such as carbon dioxide and oxygen [10,11]. The tertiary and quaternary structures of AQPs are highly conserved among taxa [12,13]. The functional unit of AQPs is organized as a tetramer, with each monomer having an independent channel consisting of an extracellular and intracellular vestibule connected by an extended narrow pore [14]. Each monomer has two main constriction sites responsible for substrate selectivity: (i) the aromatic/arginine selectivity filter (ar/R; three aromatic amino acids and one arginine) and (ii) two conserved Asn-Pro-Ala motifs (NPA) in the middle of the channel, where the positive N-terminus ends of two half helices meet the two highly conserved NPA motifs [15,16,17]. Notwithstanding these shared structural features, the overall primary structure is poorly conserved. These differences are reflected in the high diversity of the AQP family, with several new members recently discovered due to the increase in publicly available genomes and transcriptomes. The high diversification of these membrane proteins is thought to be the result of a combination of factors, such as single gene or whole genome duplications, and horizontal gene transfers [13]. Based on the evolutionary relationships and function, the existing vertebrate AQPs have been classified into four main groups: (i) orthodox aquaporins, or AQP1-like (AQP0, 1, 2, 4, 5, 6), homologues that were initially believed to transport only water; (ii) aquaammoniaporins, or AQP8-like, represented by AQP8, sometimes included in the orthodox AQPs; (iii) aquaglyceroporins, or AQP3-like (AQP3, 7, 9, 10), allowing permeation of glycerol, urea and other neutral small solutes in addition to water; and (iv) superaquaporins (also called unorthodox aquaporins), or AQP11-like (AQP11 and AQP12), characterized by a distinct evolutionary pathway, intracellular localization and a substrate selectivity that is still unclear [11,13,18,19,20]. AQP1, 3, 5, 8, 9 and 11 also allow transport of hydrogen peroxide, and, for this biophysical feature, are called peroxiporins [21]. Certainly, the diversification of AQPs appears to depend on the taxa. For instance, the majority of bacteria present a single *Aqp* gene or just one orthodox *Aqp* plus one aquaglyceroporin, while peculiar *Aqp* paralogs were retrieved only in plants (i.e., PIPs, NIPs, TIPs, SIPs, XIPs and HIPs). In invertebrate taxa, AQPs have already been characterized in arthropods [22,23,24,25], nematodes [26] and mollusks [27,28]. It is currently assumed that invertebrate AQPs are highly similar to vertebrate AQP1 and AQP4 [29]. Thus, it is becoming evident that rapid evolutionary turnover of gene duplications and gene losses are widespread [13], supporting the role of these proteins in the evolutionary adaptation of animals and plants from environmentally dynamic areas, such as estuaries.

To date, the presence of functional AQPs in annelids has only been indirectly suggested by assessing the change in body weight of the nereidid polychaete *Perinereis* sp. with hormones stimulating water transport (i.e., angiotensin II and angiotensin III) in the presence or absence of tetrachloroaurate (III), an AQP blocker [30]. Moreover, three unpublished *Aqp* sequences of terrestrial clitellates (Oligochaeta; AC number: CAX48970.1, CAX48991.1 and CAX48991.1) are available in the nucleotide nr database and have been analyzed in a wide phylogenetic study of all prokaryotic and eukaryotic *Aqps* [13]. Annelids are an ecologically and morphologically diverse group among marine invertebrates, and their members occupy a wide range of environments and show diverse lifestyles. As indicated by recent phylogenetic analyses [31], which disfavors the further usage of the nonmonophyletic Polychaeta, most annelid diversity is comprised of two monophyletic groups, Sedentaria and Errantia, which are named after the predominant mobility behavior of their members. Such recent annelid phylogenetic research highlights the diversity of annelid body plans, and therefore these worms may be seen as good models to understand the relation between physiology, behavior and evolution of Bilateria in general. In this context, comparative studies on *Aqps* may be one way to investigate the macroevolutionary transition between annelids and related invertebrate taxa.

Scarce taxon sampling in phylogenetic analyses may hide the real abundance and diversity of *Aqps* among invertebrates. The high diversity of AQPs and their reported function in osmoregulation among plants and many vertebrates [11] lead us to speculate that annelid AQPs may have a similar function in relation to salinity variation, to which this invertebrate phylum undergoes in estuarine systems. The involvement of AQPs in annelid osmoregulation may also be related to the ability of some AQPs to mediate transmembrane transport of ammonia and urea [32,33], catabolites often exploited to maintain the osmotic balance [34]. The physiological significance of AQPs in annelids may be greater than just being involved in osmotic homeostasis. Vertebrate AQPs have pleiotropic relevance by intervening in several other biological processes such as metabolism and energy balance, cell proliferation and differentiation, tissue repair, cell migration, inflammation, circadian rhythm and aging [35,36,37,38].

In this work, we first searched and annotated *Aqp* sequences of annelids in public nucleotide, genomic and transcriptomic databases/datasets, thus identifying sequences not previously recognized as *Aqps*. Moreover, we analyzed the diversity of *Aqp* genes in some specimens of the estuarine species *Alitta succinea* (Annelida: Nereididae), by RT-PCR and sequencing. This species, usually associated with organogenic detritus and human-made substrates [39], was chosen because of its worldwide occurrence and salinity tolerance. Finally, we reconstructed the evolutionary history of annelid *Aqps* in the metazoan framework, by providing gene/transcript sequences of annelid *Aqps* and inferring their phylogenetic relationships.

## 2. Materials and Methods

### 2.1. Database Mining and Phylogenetic Analyses

*Aqp* sequences of annelids were identified through text search and BlastP/tBlastn analyses [40] in the following:

(1) the protein database of NCBI (accessed between 8 January 2019 and 21 September 2021);
(2) the nonredundant nucleotide (nr) and the Transcriptome Shotgun Assembly (TSA) databases available at NCBI (accessed between 8 January 2019 and 21 September 2021). In the related tBlastn analyses, a threshold of 1 × 10^−15^ for the e-values and 40% of query coverage was used;
(3) the two annelid genomes of *Capitella teleta* Blake, Grassle and Eckelbarger, 2009 (v.1.0 of 23 August 2007) and *Helobdella robusta* Shankland, Bissen & Weisblat, 1992 (v.1.0 of 20 September 2007) available on the JGI Genome Portal (accessed between 29 November 2018 and 11 January 2019 [41]);
(4) the PdumBase transcriptome database of *Platynereis dumerilii* (Audouin and Milne Edwards, 1833) available at http://140.109.48.81/platynereis/controller.php?action=home (accessed 11 March 2019) [42];
(5) the transcriptome of *Alitta succinea* available in the supplementary material (available at http://dx.doi.org/10.5061/dryad.30k4v) of Kocot and coworkers [43];
(6) the *Aqp* dataset of metazoans analyzed in Abascal and coworkers [13]. Sequence searches were not performed in the SRA (Sequence Read Archive) database (accessed 29 November 2020) to avoid necessary accurate assembly protocols that were not in the scope of this study.

In BlastP and tBlastn searches (accessed between 8 January 2019 and 21 September 2021), we used as queries the amino acid sequences of all known human AQPs, and those of the annelid *Aqps* that were progressively identified. In the genomic databases of *C. teleta* and *H. robusta*, text search was performed by searching for gene sequences with hits to protein domain database entries describing the AQP family (i.e., hits to the entries PF00230-“Major intrinsic protein” of Pfam [44], IPR000425-“MIP” of Interpro [45], and KOG0223-“Aquaporin (major intrinsic protein family)” of CDD [46]. Indeed, no one of the identified *C. teleta* and *H. robusta Aqps* was annotated as such in the genomic database.

Accession numbers or reference sources of all selected sequences are listed in the Supplementary Materials (Table S1).

The 18 *Aqp* transcripts identified in the *A. succinea* transcriptome were compared one to the other with Geneious Pro ver. 5.5.7.2 (http://www.geneious.com), to find overlapping sequence regions and to reconstruct the longest sequences putatively corresponding to distinct genes. Redundant transcripts (i.e., identical sequences of the same species), retrieved from the TSA databases were identified and eliminated in Geneious Prime Pro ver. 5.5.7.2 (http://www.geneious.com).

*Aqp* sequences were also searched in the available genomic and transcriptomic sequences of 31 representative species of 27 additional metazoan phyla (see Supplementary Table S2 for species list and analyzed databases). For these analyses, tBlastn searches were performed using several *Aqp* sequences selected among the taxa evolutionarily closest to the species under investigation as queries. No genomes/transcriptomes were found for the phyla Onychophora and Kynorincha.

Multialignment of the selected amino acid sequences was carried out with MUSCLE [47] as implemented in SeaView X [48], and it was manually trimmed and optimized. Phylogenetic inferences were performed using the overall amino acid multialignment containing all metazoan sequences and on a reduced version including only annelid *Aqps*. Metazoan and annelid amino acid multiple alignments of *Aqp* sequences are available in Supplementary Data S1 and S2 (full-length) and S3 and S4 (trimmed), respectively. Maximum likelihood (ML) trees were inferred using PhyML (accessed 23 October 2021) [49], and the robustness of nodes was estimated using an approximate likelihood ratio test (aLRT) [50]. The best amino acid substitution model was selected with the SMS routine in PhyML using both AIC and BIC as optimality criteria [51]: LG + G_4_ + F was selected for the metazoan alignment, and VT + G_4_ + F was selected for the annelids. Bayesian inference (BI) analysis was performed using BEAST 2.6.4 [52]. The site model was set up according to SMS selection. The tree prior was set to Birth-Death. No molecular calibration was applied, leaving strict clock at rate of 1 as prior. Four runs of Markov chain Monte Carlo (MCMC) were performed each 400 M generations long, sampled every 20,000 generations. Runs were examined for convergence in Tracer 1.7. All parameters of runs reached the effective sample size (ESS) above 100 and were combined using LogCombiner 2.6.4, to obtain ESS above 200. Burn-in phase of 30% was removed from each run. The final tree was summarized from combined runs with TreeAnnotator 2.6.4.

The final trees were edited with FigTree 1.4 [53]. In accordance with previous phylogenetic analyses, these trees were re-rooted using aquaglyceroporins, also named AQP3-like clade, which formed a highly supported monophyletic group [13,54].

### 2.2. Specimens Collection

Specimens of the estuarine *A. succinea* were obtained from Lesina Lake, located on the coast of the southwestern Adriatic Sea, Italy. The lake is subjected to marked salinity variations due to limited connections with seawater and periodic freshwater inputs, with a salinity range of 11–34 psu and a temperature range of 7–26 °C depending on the season [55,56]. Twenty specimens were collected from mud sediment using an Ekman grab. In the laboratory, animals were acclimated for 48 h in two plastic boxes of 1 L lake water under constant temperature (~20 °C), salinity (~10 psu), aeration and natural photoperiod. Ten animals were acclimated in each box, one containing Lesina Lake sediment as a food source (fed animals), and the other without sediment/food (starved animals). Depending on their size, two/three individuals of either fed or starved animals were then aliquoted in separate cryogenic vials, flash frozen in liquid nitrogen and stored at −80 °C until RNA extraction.

### 2.3. RNA Extraction, Aqps Cloning and Sequencing

Total RNA was extracted from the central portion of the body of two/three frozen *A. succinea* worms (~100 mg of tissue) from either fed or starved individuals, using TRIzol (Invitrogen, Carlsbad, CA, USA), following the manufacturer’s protocol. Multiple RNA extractions were carried out. Reverse transcription of total RNA was performed with Super Transcript III (Thermo Fisher Scientific, Carlsbad, CA, USA) in accordance with the manufacturer’s instructions. First-strand cDNA synthesis was performed using mixed random primers and oligo(dT) primers. Amplifications of *Aqp* cDNA were carried out using specific primers (Supplementary Table S3) manually designed on the *Aqp* transcripts identified in the *A. succinea* transcriptome of Kocot and coworkers [43]. Amplifications were performed in a 25 µL reaction volume containing 20 mM PCR buffer, 0.5 µM forward and reverse primers, 0.2 mM of each dNTP, 1.25 U/µL DreamTaq (Thermo Fisher Scientific, Carlsbad, CA, USA), 3–8 µL of template cDNA and nuclease-free water to bring the mix to a final volume of 25 µL. The amplification conditions were as follows: an initial denaturation for 3 min at 95 °C, then 30 amplification cycles (denaturation for 30 s at 95 °C; annealing for 30 s at 54–56 °C depending on the primer pair; extension for 2 min at 72 °C) followed by a final elongation step of 10 min at 72 °C (Supplementary Table S3). The amplicons obtained were cloned with TOPO TA cloning kit (Thermo Fisher Scientific, Carlsbad, CA, USA). Positive clones were sequenced according to the Sanger method at Eurofins Genomics (Ebersberg, Germany) or Microsynth AG (Balgach, Switzerland). Sequence quality checks, assembly and comparisons to the gene annotations were carried out with Geneious Prime Pro ver. 5.5.7.2 (http://www.geneious.com). The produced sequences were submitted to GenBank with Accession numbers OL752693-96.

### 2.4. Three-Dimensional Structural Predictions

The tertiary structure of *A. succinea Aqps* was predicted using Phyre2 (accessed 15 December 2020) [57], considering only the best hit models with a confidence of 100% and a coverage of at least 80%. Cartoon renderings of the proteins were produced in EzMol (accessed 23 December 2020) [58].

## 3. Results and Discussion

**Figure 1:**
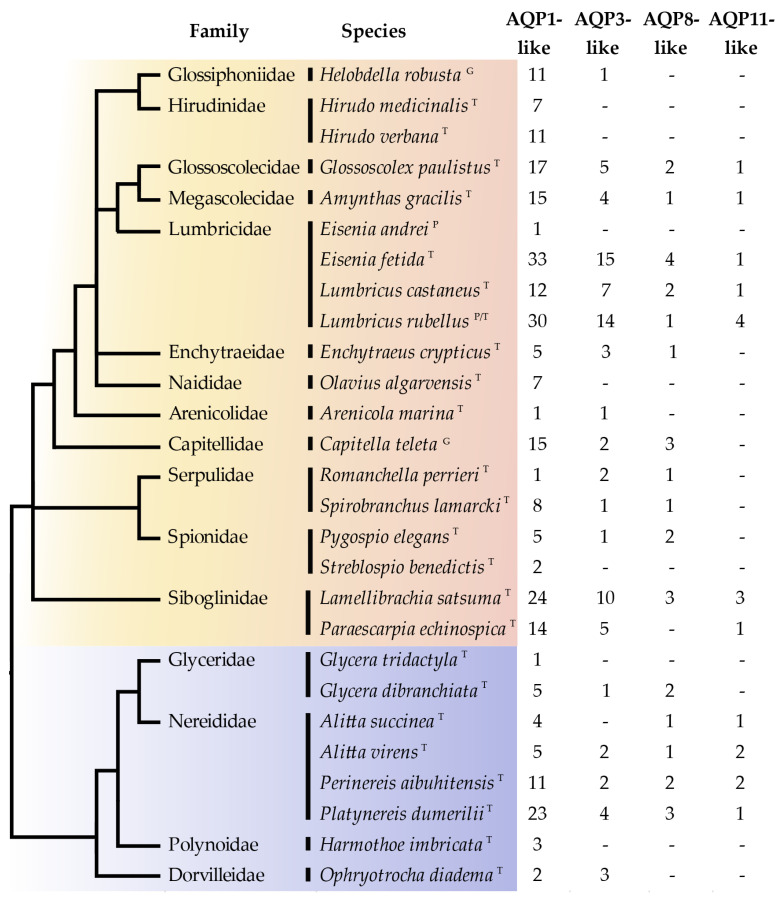
Evolutionary distribution of the annelid *Aqps* identified in this study, based on amino acid data. Annelid phylogenetic tree derived from the work in [31] and was combined with the Clitellata tree adapted from in [59]. Annelids belonging to Errantia are in blue (below) while families belonging to Sedentaria are in orange (above). *Aqp* classification according to phylogenetic relationship recovered in Figure 2. Letters following the species indicate the source of the analyzed data: G = genome; T = transcriptome; P = protein.

We identified a total of 401 annelid *Aqps* (summarized in Figure 1) from 27 annelid species, belonging to 16 families (i.e., 12 Sedentaria and four Errantia). Only three of these sequences were annotated as *Aqps* in the widely used nr database of NCBI (they were also present in the Abascal and coworkers *Aqp* phylogenetic reconstruction [13]). On the contrary, all *Aqps* identified in the transcriptome assemblies were indicated in the TSA database only as “transcribed RNA”, and the CDS (CoDing Sequence) annotation was also missing. The *Aqps* identified in the JGI genomic databases were only partially annotated, since the description line was empty; thus, they can be retrieved only searching for hits to the AQP-specific conserved domain. The distribution of the identified annelid sequences among the different *Aqp* groups indicates that no major *Aqp* clade previously found in other invertebrates/vertebrates was absent in the two main annelid groups of Errantia and Sedentaria (Figure 1). Moreover, the AQP1-like group is the most abundant one in annelids, similar to what was observed in vertebrates, see, e.g., in [13]. Numbers in Figure 1 represent only a rough estimate of the number of *Aqp* genes or transcripts present in each species. Indeed, on one hand, the number of genes inferred from genomic data could have been underestimated due to partial or low-quality genome assembly. On the other hand, the number of transcripts inferred from transcriptome data could have been affected by the presence of alternative splicing isoforms and by the possible low or tissue-specific expression of some *Aqp* genes. Finally, our similarity search approach could have hampered the identification of possible fast-evolving (and therefore highly divergent) annelid-specific *Aqps.*

**Figure 2:**
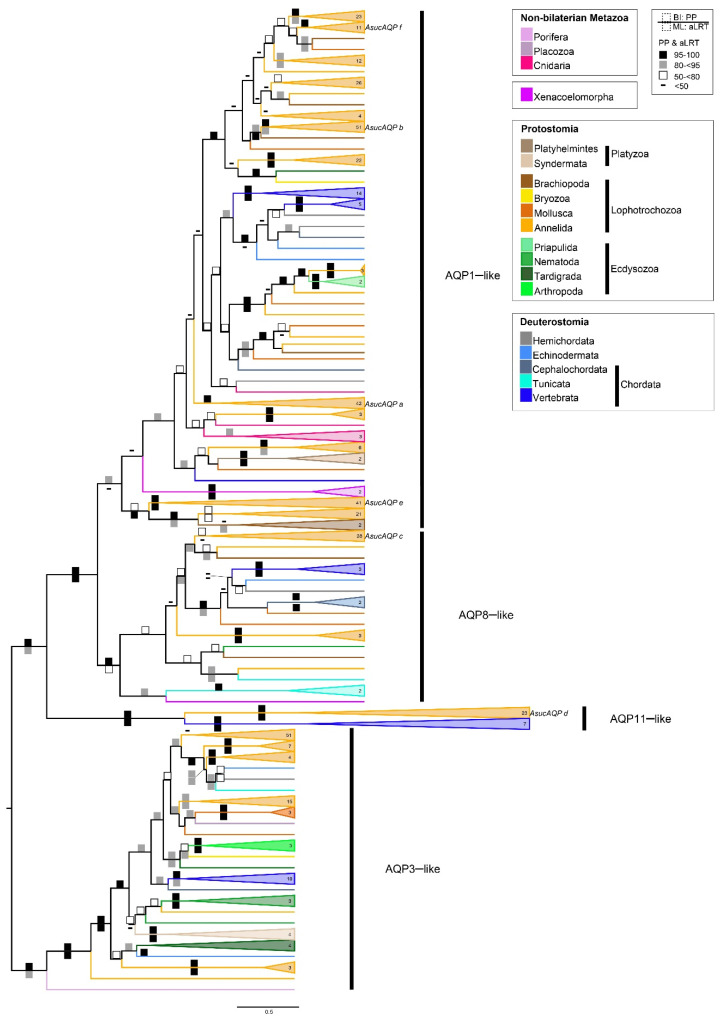
Phylogenetic reconstruction of metazoan *Aqps*. Bayesian tree obtained from 518 sequences, 418 amino acid sites, re-rooted with AQP3-like group. Squares on branches represent the support values for Bayesian inference (BI posterior probabilities; upper square) and maximum likelihood (ML aLRT; lower square) reported as percentages (nodes not recovered in the ML are without symbols). Branches are coloured according to taxonomy. Labels in the tree refer to the annelid *Alitta succinea Aqps* (*AsucAQPa, b, c, d, e, f)* described in Table 1. The fully annotated and midpoint-rooted trees, the alignment and analyzed sequences are available in the Supplementary Materials (Figures S1 and S2; Data S1; Table S1).

A collapsed version of our *Aqp* phylogenetic reconstruction of metazoans is shown in Figure 2, whereas fully annotated and midpoint-rooted trees are provided in the Supplementary Materials (Figures S1 and S2). This tree recognizes three well-supported clades, named AQP3-like, AQP8-like and AQP11-like, in accordance with the vertebrate AQPs they contain, and the nomenclature of Soto and coworkers [18]. A fourth clade, named AQP1-like, is marginally supported only by the Bayesian tree. Excluding the AQP11-like group, the phylogeny of each *Aqp* main group generally reflects the current metazoan tree [60,61], with poriferans and cnidarians as sister groups of Bilateria, and Xenacoelomorpha as sister group of Nephrozoa (i.e., bilaterians with nephridia). The latter is divided into two clades: Protostomia, with annelids, mollusks, bryozoans and brachiopods branching within Lophotrochozoa, and Deuterostomia (Figure 2). No *Aqp* genes were retrieved in 11 of the 27 additional metazoan phyla analyzed here (i.e., Ctenophora, Chaetognata, Gnathostomulida, Micrognathozoa, Gastrotricha, Phoronida, Entoprocta, Cycliophora, Rhombozoa, Nematomorpha and Loricifera, see Supplementary Table S2).

The AQP11-like clade is characterized by a very long branch and consists of two highly supported subclades, one including only annelids, and the other only vertebrates (Figure 2). Moreover, the annelid subclade consists of only a few families of both Sedentaria and Errantia groups (i.e., the families Glossoscolecidae, Megascolecidae, Lumbricidae, Siboglinidae and Nereididae, see Supplementary Figures S1 and S2). Therefore, we cannot exclude that the AQP11-like clade is an artefactual clade due to long branch attraction between fast-evolving sequences of vertebrates and annelids.

**Figure 3:**
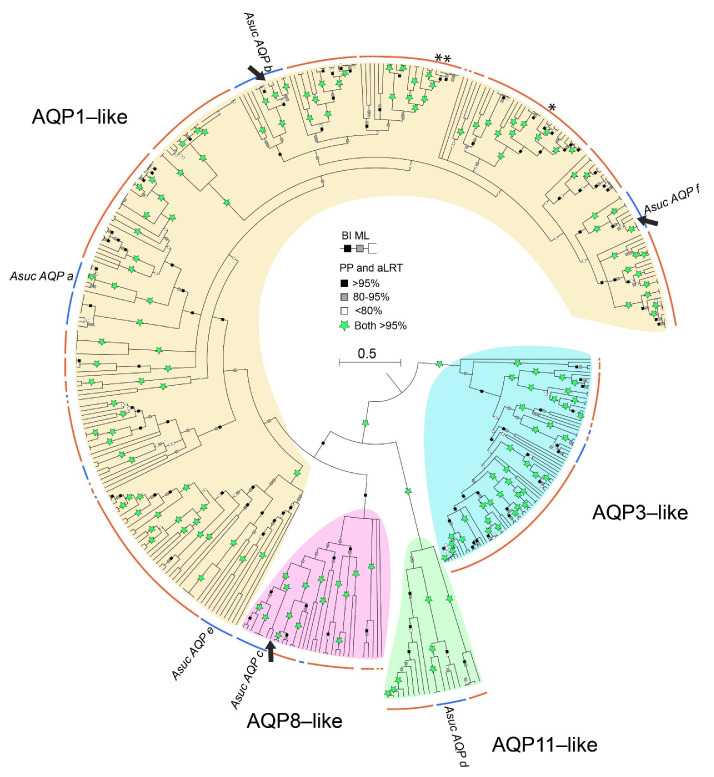
Phylogenetic reconstruction of annelid *Aqps*. Bayesian tree obtained from 401 sequences, 461 amino acid sites, re-rooted with AQP3-like group. Squares on branches represent the support values for Bayesian inference (BI posterior probabilities; upper square) and maximum likelihood (ML aLRT; lower square) reported as percentages. Nodes < 50% PP collapsed. Labels in the tree refer to the annelid *Alitta succinea Aqps* (*AsucAQPa, b, c, d, e, f*). Arrows indicate the sequenced *AsucAQPs*. The asterisk indicates the only three annelids analyzed in [13]. Orange and blue curved lines refer to Sedentaria and Errantia groups, respectively. The fully annotated trees, the alignment and the analyzed sequences are available in the Supplementary Materials (Figures S3 and S4; Data S2; Table S1).

Besides the AQP11-like clade, almost all annelid clades are sister to lophotrochozoan species/clades (Figure 2 and Supplementary Figures S1 and S2), with a few exceptions characterized by a polytomy or low statistical support of the immediately previous node (e.g., the clade containing *AsucAQPa*). This could indicate that the absence of gene duplications occurred exclusively in the annelid ancestor, except for the ambiguous result of the clade of *AsucAQPa*, which contained only annelids. On the contrary, several gene duplications seem to have occurred in the ancestor of some Sedentaria families (e.g., Lumbricidae, Megascolecidae, Glossoscolecidae and Spionidae), especially in the AQP1-like clade (Supplementary Figures S1–S4). For instance, this can be observed in the clade containing *AsucAQPe* (Figure 2 and Figure 3, Supplementary Figures S3 and S4), consisting of a single Errantia cluster grouped to a large Sedentaria one with duplicated subclades of 2–3 taxonomic families; or the Sedentaria clade, sister to the Errantia one containing *AsucAQPf*, in which two rounds of duplications probably occurred in the common ancestor to Lumbricidae, Megascolecidae and Glossoscolecidae.

A collapsed version of the annelid *Aqp* tree is shown in Figure 3, whereas the fully annotated trees are provided in Supplementary Figures S3 and S4. This tree (based on 401 sequences) generally recovered the clades of Sedentaria and Errantia well separated as in the recent literature [31,59]. Clades AQP3-like and AQP11-like showed high support in both BI and ML. Most of the sequences found (271) belonged to the AQP1-like clade, showing multiple paralogs for each species. Even the three terrestrial Oligochaeta analyzed in a previous wide *Aqp* phylogenetic study [13] were also included in the AQP1-like clade (see asterisks in Figure 3). Noteworthy, many clades were exclusive to Sedentaria, suggesting the occurrence of several distinct gene duplication events in the ancestor of Sedentaria. The presence of numerous subclades with high support (>95%) in the AQP1-like clade of annelids, reflects what has been observed in other invertebrates [13,27]. The structure, function and body localization of AQPs of insects and mollusks have already been recognized as very similar to those of vertebrates AQP1 and AQP4, leading to the hypothesis of an orthologous relationship [27,29]. The similarity between invertebrate AQP1-like and vertebrate AQP1/AQP4 is also supported by the wide distribution of vertebrates AQP1 and AQP4 along the animal bodies and by their more basal position in several vertebrate clades of orthodox AQP.

**Figure 4:**
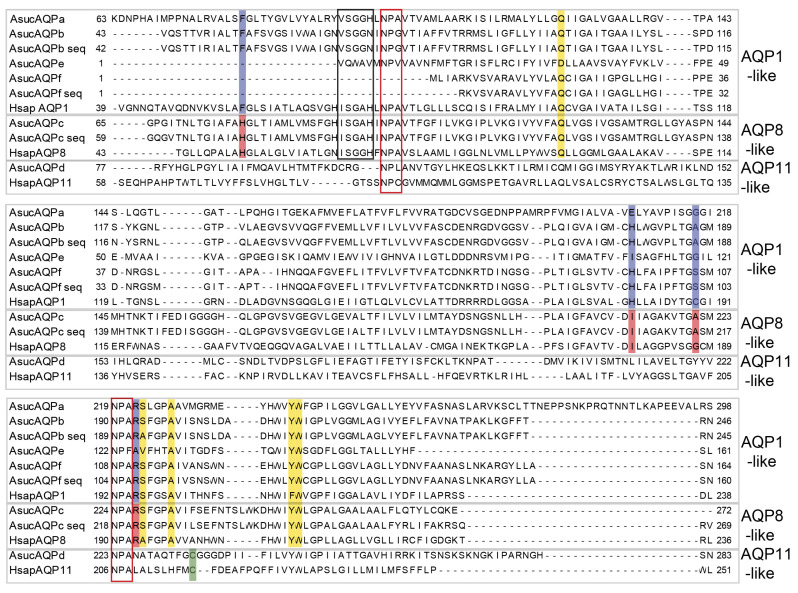
Multiple alignment of the *Alitta succinea Aqps* (Table 1) and human (Hsap) AQP1 (P29972), AQP8 (XP_011544124.1) and AQP11 (Q8NBQ7). The “seq” suffix marks sequences obtained by RT-PCR, cloning and sequencing. In red boxes the two NPA motifs. The black box indicates the ISSGH motif, typical of the AQP1-like clade. The ar/R regions of both AQP1-like and AQP8-like clades are shaded in blue and red, respectively. The Cys residue down in the C-terminus NPA boxes of the AQP11-like clade is shaded in green. Positions P1–P5 of the AQP1-like clade are shaded in yellow.

We retrieved and annotated a total of six *Aqp* genes from the transcriptome of *A. succinea* published by Kocot and coworkers [43] and named them AsucAQP from a to f (Table 1 and Figure 4). These six paralogs clustered with three of the four main AQP groups of vertebrates (Figure 2 and Figure 3). Indeed, *AsucAQPa, b, e* and *f* cluster into the AQP1-like clade; *AsucAQPc* is inside the AQP8-like clade; and *AsucAQPd* belongs to the AQP11-like clade. We managed to confirm the sequence of three of these *A. succinea Aqps* by RT-PCR and sequencing, namely, *AsucAQPb, AsucAQPc* and *AsucAQPf*, but these cDNA amplifications occurred only in fed animals (Table 1). The starvation faced by some of our *A. succinea* specimens, coupled with their potentially different life stages, may have influenced the extent of expression of *Aqps*, and could explain why we were able to amplify these *Aqps* by RT-PCR only in fed animals [42,62]. Moreover, two mRNA isoforms of *AsucAQPb* were sequenced, differing only in the 3’UTR (see Accession Numbers OL752695-6). Few differences were found in the amino acid sequences between the three annotated transcripts *AsucAQPb, c*, and *f* from Kocot and coworkers [43] and our corresponding sequenced cDNAs (Figure 4). Indeed, we observed seven amino acid differences (95.86% identity) in *AsucAQPb*, 24 (95.08% identity) in *AsucAQPc* and seven (95.86% identity) in *AsucAQPf* (Figure 4). These differences can be easily explained by the intraspecies variability of *A. succinea*, as they do not occur in evolutionarily invariant amino acid positions of *Aqps*.

*AsucAQPa, AsucAQPb, AsucAQPe* and *AsucAQPf*, together with the majority of the protostome *Aqps* analyzed here, grouped within the AQP1-like clade (Figure 2). These *Aqps*, like most orthodox ones, present a very conserved ar/R region with a typical aromatic Phe in the first position, an ISSGH motif located upstream of the first NPA, and P1-P5 residues (Figure 4), which all play an important role in their structures and functions [27,63,64]. *AsucAQPa, AsucAQPb* and *AsucAQPe* presented the ISGGH motif with a substitution of the first residue with Val (Figure 4). Analysis of the five polypeptide positions (P1–P5) retrieved the residues Gln-Ser-Ala-Tyr-Trp, in almost all the AsucAQP1-like sequences (Figure 4). The ar/R region of these four *Aqps* displayed substitutions in the second and third residues in comparison with human AQP1 (Figure 4). The ar/R region of *AsucAQPa* was formed by the amino acids Phe, Glu, Gly and Arg, presenting a substitution in the second position compared with human AQP1, which displays a His (Figure 4). The *AsucAQPb* ar/R filter was formed by Phe, His, Ala and Arg, while human AQP1 has Cys in the third position (Figure 4). Substitutions in these more conserved regions are not infrequent in other organisms. For instance, in plant *Aqps*, such as *Arabidopsis thaliana* (L.) Heynh, or animals, such as *Homo sapiens,* ISSGH motifs present similar replacements. Moreover, the P1 residue retrieved as Gln in this study is represented by a Thr in organisms other than annelids [27,63]. Additionally, some freshwater pulmonated gastropods, such as *Lymnaea stagnalis* (Linnaeus, 1758), *Stagnicola palustris* (O.F. Muller, 1774) and *Ladislavella occulta* (Jackiewicz, 1959) show similar substitutions with amino acids with similar chemical properties (i.e., Gln instead of Thr in *LsAQP1*, *SpAQP1* and *CoAQP1*). These *Aqps*, were already classified as genes belonging to the AQP1-like clade, suggesting that these differences do not affect their water channel function [27].

*AsucAQPa* was located in a clade containing only annelids, while the other paralogs (i.e., *AsucAQP b, c, e* and *f*) clustered with other lophotrocozoan taxa. *AsucAQPe* was recovered in the lophotrochozoan clade as well, as a sister group of the remaining sequences of the AQP1-like clade (Figure 2).

Nevertheless, the existence of AQP paralogs only in some taxa is not infrequent. For instance, Maqp and Mglp are found only in mollusks [28], while Drip, Prip and Bib are retrieved only in arthropods [65,66,67]. Considering the diversity of the Lophotrochozoa clade, inhabiting marine, freshwater or terrestrial environments, with different functional traits and body plans [68], it is tempting to hypothesize that the high diversification of AQPs relates to physiological adaptations, such as osmoregulation and desiccation. Moreover, in terms of osmoregulation, unlike vertebrates, which rely on a more complex excretory system such as nephrons, Lophotrochozoa usually display different osmoprotective mechanisms, such as those implemented at the level of the epidermis, nephridia (proto- and metanephridia) and Malpighian tubules [65]. The presence of multiple substitutions in the NPA motifs supports our idea that AQPs may have roles in the adaptations to salinity variation in estuarine systems. Moreover, the NPG box in the N-terminus of *AsucAQPb* (recovered only in annelids) was previously known from some plants (in the C-terminus)**.**

Phylogenetic hypotheses placed *AsucAQPc* as the ortholog of vertebrate AQP8 (Figure 2). The ar/R region of *AsucAQPc* was formed by His, Ile, Ala and Arg, similar to the residues found in the corresponding region of mammalian AQP8 (Figure 4), an AQP featuring transport of ammonia, urea and hydrogen peroxide in addition to water [69]. The only difference was found in the third position, in which the annelid Ala is replaced by Gly in humans. The contribution of this protein to ammonia transport may be fundamental for osmoregulation, helping in regulating salt/water balance in annelids. For instance, this protein was also reported to increase the water permeability of membranes by redistributing intracellular vesicles to the plasma membrane in vertebrates [33,70,71]. Putative orthologs of AQP8 have already been found in plants (i.e., Tonoplast Intrinsic Protein (TIPs)). In fact, some authors have already debated whether the molecular similarities of these proteins were synapomorphies, supporting the hypothesis of a most recent common ancestor for both animal and plant *Aqps* [18], or if this was the result of similar functions in animals and plants that led through natural selection to sequence convergence [13].

*AsucAQPd* was retrieved in the AQPs11-like clade as a putative ortholog of vertebrates AQPs11 and AQPs12 (Figure 2), with which shared Cys in the ninth position after the second NPA motif, considered the signature of this superaquaporin group (Figure 4) [72]. Nonetheless, its presence in annelids is debatable. The long branches and a sister group position in the phylogeny considering all the non-aquaglyceroporins reflect the high divergence of vertebrate superaquaporins from the other AQPs in terms of structure and function. Their similarity with intracellular SIPs, an *Aqp* clade typical of plants, and a potential ancestral gene common to both clades was suggested [18]. However, when analyzing the sequences of SIPs, no conserved residues with AQP11 were found, pointing out that the association within the same clade is more likely a long branch artifact rather than a real relationship of orthology [13]. On the other hand, high variability in the overall *Aqp* primary structures and substitutions within the NPA motifs is common in invertebrates [73], thus potentially explaining the great difference of our *AsucAQPd* compared with the already known vertebrate AQP11 and AQP12. Moreover, other studies have also suggested the presence of orthologs of these vertebrate AQPs in mollusks, arthropods and nematodes [28,66,74].

No aquaglyceroporins were recovered in the *A. succinea* transcriptome; however, their presence was retrieved in the congeneric *A. virens* (Sars, 1835)*,* as well as in the other annelids and invertebrates (Figure 2 and Figure 3) [66,73,75]. Their presumed absence may be due to low sequencing depth (e.g., only one sample [43]) which may have resulted in a limited number of genes recovered in the transcriptome. Indeed, the presence of aquaglyceroporins was associated with a change in membrane water permeability during hypoosmotic stress [76,77]. For instance, the invertebrate *Caenorhabditis elegans* (Maupas, 1900) (Nematoda) showed the involvement of an aquaglyceroporin (therein called *Aqp-8*) in response to either hyper- or hypoosmotic stress by increasing vesicle docking to the lumen of excretory cells and promoting the transport of water and osmotically active solutes to maintain intracellular homeostasis [78]. The importance of these water channels is also testified by their presence in a wide range of taxa from bacteria and archaea through all domains and kingdoms, see, e.g., in [13,74,79].

**Figure 5:**
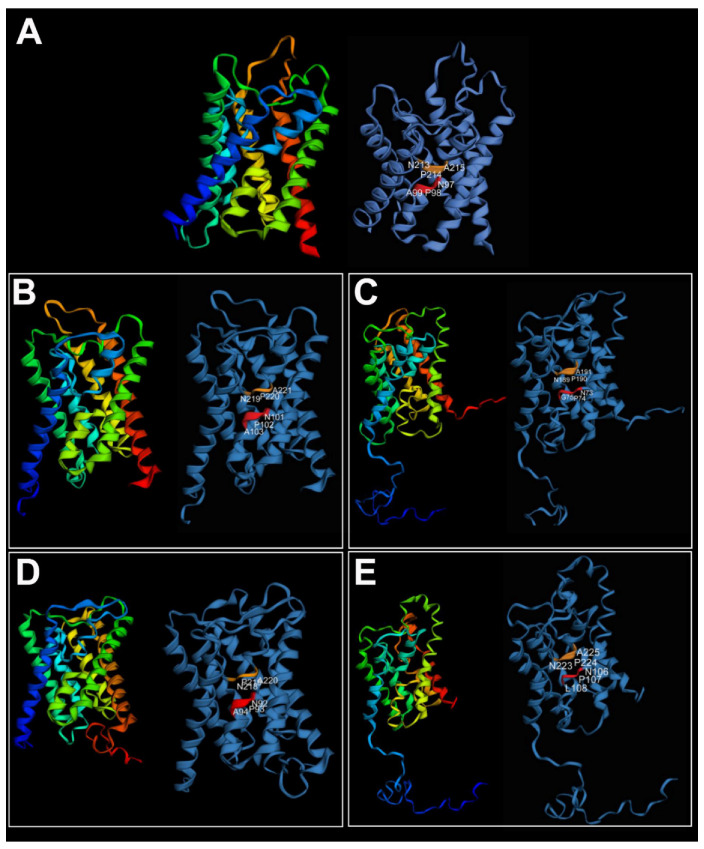
Three-dimensional structure of (**A**) *Homo sapiens* AQP4 (PDB ID: 3GD8) used as template; (**B**) *AsucAQPa*; (**C**) *AsucAQPb*; (**D**) *AsucAQPc*; (**E**) *AsucAQPd*. On the left, AQP structures colored in rainbow indicate N to C termini, from blue to red. On the right, NPA motifs are indicated in red and orange. The three-dimensional structure was predicted only for *AsucAQPs* with complete or almost complete coding sequences.

High similarity among the tertiary structures of the *A. succinea Aqp* genes was recovered when compared with the vertebrate AQP4. *Alitta succinea Aqps* consisted of six transmembrane domains plus two additional membrane-embedded α-helices, displaying the typical hourglass shape of AQPs (Figure 5).

Finally, the presence of more than one paralog in several annelid species, especially in the AQP1-like clade, may be the result of multiple recent gene duplications. These events are common in adaptive processes and may lead to co-option and to a consequently functional diversification of the genes through changes in its regulatory levels and/or in parts of the amino acid sequence not required for the current function [80]. This is the case of the Eglp of some insects (i.e., Holometabola), ortholog to vertebrate AQP4, which evolved in a glycerol facilitator, after a single mutation of His174 from the ar/R region with Ala174 [81].

## 4. Conclusions

The widespread presence and abundance of AQPs are the result of their relevance in a variety of biological processes, such as body water homeostasis and metabolism. We reconstructed, for the first time, the evolutionary history of annelid *Aqps*, and confirmed by RT-PCR/sequencing three of the six *Aqps* annotated in *Alitta succinea*, an estuarine annelid showing high salinity tolerance. Our findings are congruent with previous studies concerning the evolution of *Aqps* across metazoans [13,18]. Our results support the existence of four major *Aqp* clades (AQP1-like, AQP8-like, AQP11-like and AQP3-like), and the hypothesis of the existence of four ancestral genes, one for each of the four AQP-like clades, in the common ancestor of invertebrates and vertebrates. Moreover, a general trend towards taxon-specific gene duplications in annelids, implying the expansion of the AQP1-like clade, is also visible. Finally, we inferred the physiological roles of annelid *Aqps* on the basis of models already known or applied to vertebrates. Further studies on annelid AQPs are highly advisable to explore how different combinations of amino acids along their primary sequence may influence their transport selectivity. We believe that our data could stimulate and address future analyses on the expression regulation, physiological significance and functional role of AQPs in annelids, as well as provide important insights into the understanding of the osmoregulation and cell volume homeostasis of these soft-body animals marked by successful adaptive radiation.

## Figures and Tables

**Table 1:** The six *Aqp* transcripts annotated in the transcriptome of *A. succinea* published by Kocot and coworkers [43]. Asterisk: *Aqps* amplified by RT-PCR and sequenced; CDS: coding sequence, corresponding to the ORF; np: not present.

Sequence	5′ UTR	CDS	3’ UTR	CDS Notes	Total bp
*AsucAQPa*	373	999	261		1633
*AsucAQPb* *	27	804	447		1278
*AsucAQPc* *	141	818	634		1593
*AsucAQPd*	248	922	np	3′ partial	1170
*AsucAQPe*	np	504	np	5′ and 3′ partial	504
*AsucAQPf* *	np	584	324	5′ partial	908

## Data Availability

The data presented in this study are available in Supplementary Materials.

## Supplementary Materials

The following are available online at https://www.mdpi.com/article/10.3390/cells10123562/s1. Table S1: Species, accession numbers, AQP clade; Table S2: List of Non-annelid species used in analyses; Table S3: Primer list and relative PCR thermal conditions; Data S1: Alignment metazoans; Data S2: Alignment annelids only; Data S3: Trimmed alignment metazoans; Data S4: Trimmed alignment annelids only; Figure S1: Metazoan Bayesian tree fully annotated; Figure S2: Metazoan PhyML phylogenetic reconstruction fully annotated; Figure S3: Annelids only Bayesian tree fully annotated; Figure S4: Annelids only PhyML phylogenetic reconstruction fully annotated.
