# Supplementary figures and images for: Finding Aquaporins in Annelids: An Evolutionary Analysis and a Case Study
Source: Cells. 2021 Dec 17;10(12):3562. doi: 10.3390/cells10123562 (PMC8700629; doi:10.3390/cells10123562)

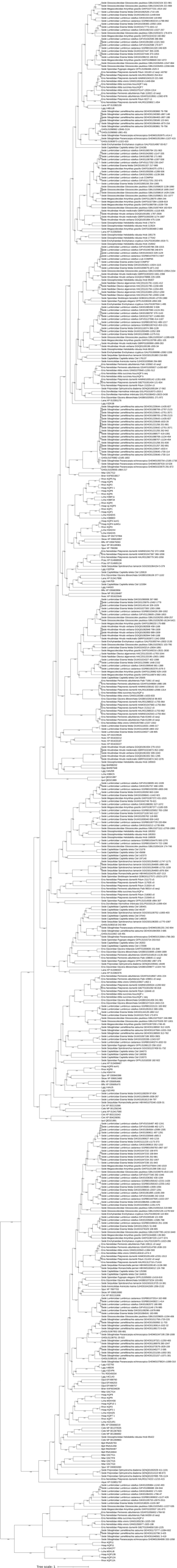

Supplement: Supplementary file 1 [file cells-10-03562-s001.zip › Suppl_Figure S1_ Metazoan bayesian tree fully annotated. Posterior probabilities above each branch.pdf]

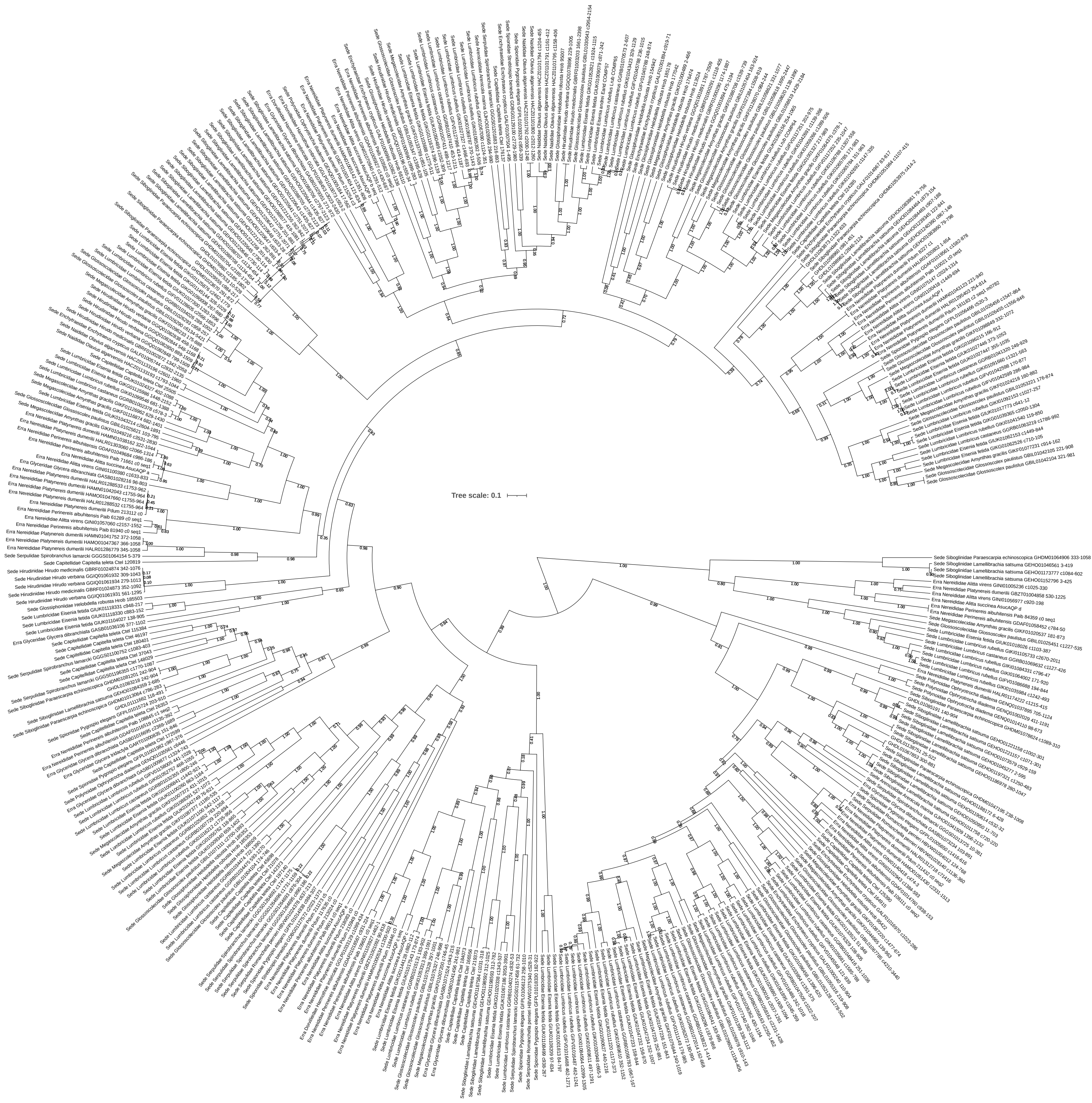

Supplement: Supplementary file 1 [file cells-10-03562-s001.zip › Suppl_Figure S3_ Annelids only bayesian tree fully annotated.pdf]

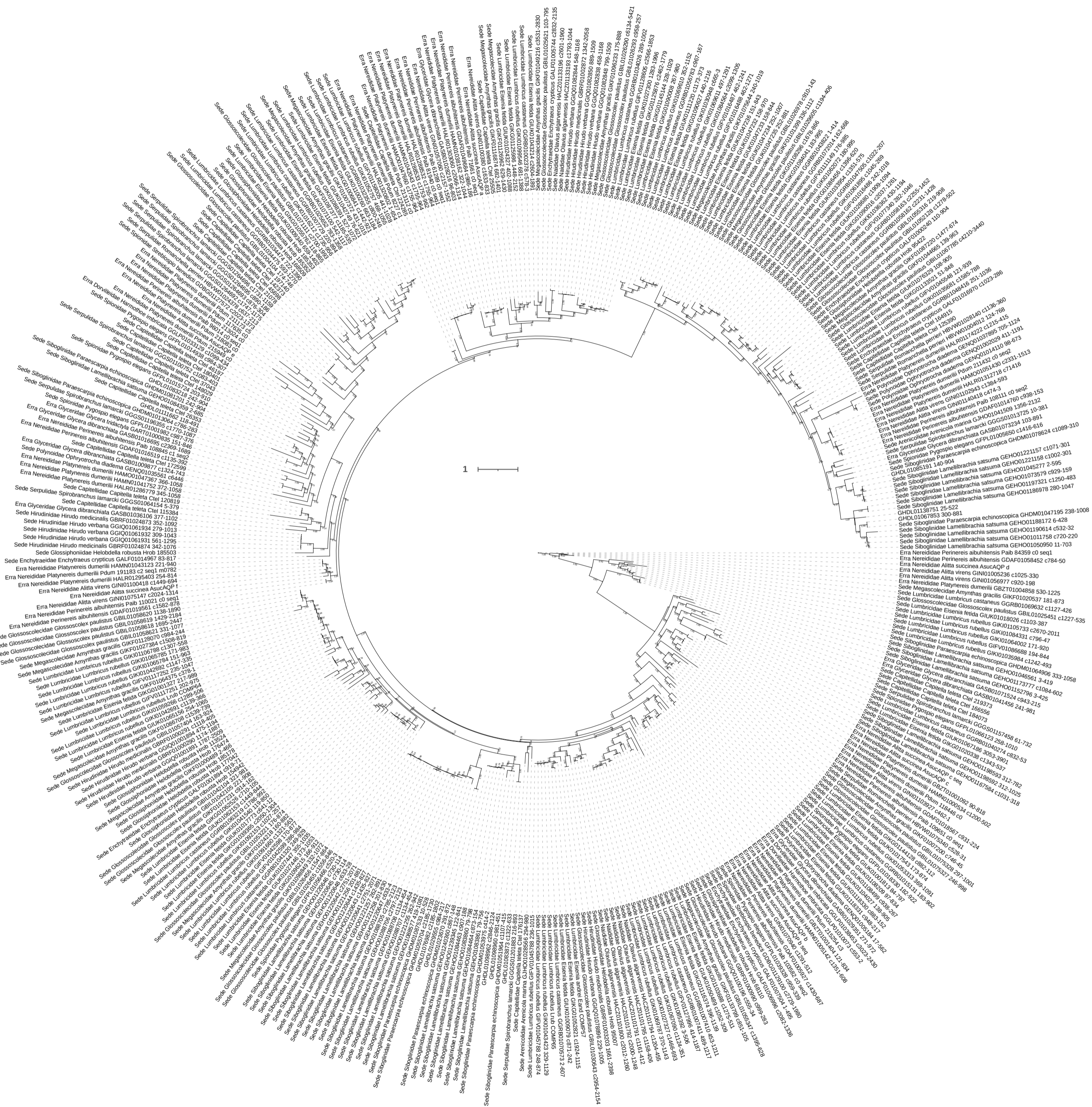

Supplement: Supplementary file 1 [file cells-10-03562-s001.zip › Suppl_Figure S4_Annelids only PhyML fully annotated.pdf]
