# Supplementary material for: Finding Aquaporins in Annelids: An Evolutionary Analysis and a Case Study
Source: Cells. 2021 Dec 17;10(12):3562. doi: 10.3390/cells10123562 (PMC8700629; doi:10.3390/cells10123562)

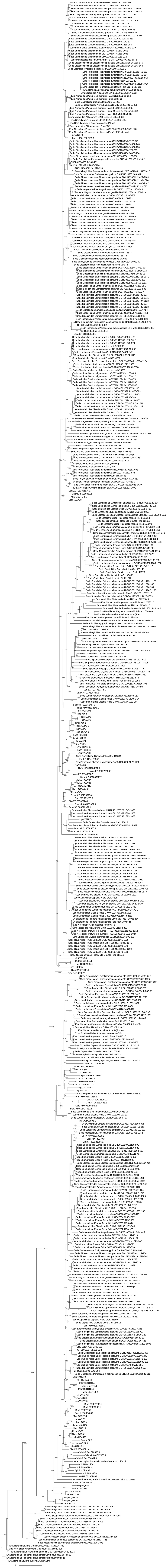

Supplement: Supplementary file 1 [file Suppl_Figure S2_ Metazoan Phyml phylogenetic recostruction fully annotated. ALRT support values on the branches.pdf › Suppl_Figure S2_ Metazoan Phyml phylogenetic recostruction fully annotated. ALRT support values on the branches.pdf]
